
JOURNAL INFORMATION
==============================
NLM Title Abbreviation: ScientificWorldJournal
Journal ID: ScientificWorldJournal
NLM Journal ID: 101131163
Journal ID: TSWJ

The Scientific World Journal

ISSN: 2356-6140
EISSN: 1537-744X
Publisher: Wiley

ARTICLE INFORMATION
==============================
PMCID: PMC6084545
PMID: 30147524
DOI: 10.1100/tsw.2001.173
Article ID: 676917
Article version: 1
Subjects: Short Report

Secondary Necrosis of Apoptotic Neutrophils Contributes to Inflammatory Lung Injury in vivo

Medan Djordje 1
Wang Liying 2
Rojanasakul Yon 1 *
West Virginia University, Department of Basic Pharmaceutical Sciences, Health Sciences Center, P.O. Box 9530, Morgantown, WV 26506, USA
National Institute for Occupational Safety and Health, Pathology and Physiology Research Branch, Morgantown, WV 26505, USA
*Yon Rojanasakul: yrojanasakul@hsc.wvu.edu
Publication date: 2001
Electronic publication date: 2001 Apr 4
Volume: 1
First page: 57
Last page: 57
Copyright: Copyright © 2001 Djordje Medan et al.
Copyright year: 2001
License: This is an open access article distributed under the Creative Commons Attribution License, which permits unrestricted use, distribution, and reproduction in any medium, provided the original work is properly cited.
License URL: https://creativecommons.org/licenses/by/3.0/

Funding: National Institutes of Health HL62959

==============================
Miami *Nature Biotechnology* Short Reports
*TheScientificWorld* (2001) 1(S3), 57SR
ISSN 1532-2246; DOI 10.1100/tsw.2001.173

### SECONDARY NECROSIS OF APOPTOTIC NEUTROPHILS CONTRIBUTES TO INFLAMMATORY LUNG INJURY *IN VIVO*

Djordje Medan[1], Liying Wang[2], and Yon Rojanasakul[1]*

[1]West Virginia University, Department of Basic Pharmaceutical Sciences, Health Sciences Center, P.O. Box 9530, Morgantown, WV 26506, USA; [2] National Institute for Occupational Safety and Health, Pathology and Physiology Research Branch, Morgantown, WV 26505, USA
* yrojanasakul@hsc.wvu.edu

**INTRODUCTION**. Apoptosis has been suggested as a means to facilitate the resolution of pulmonary inflammation (1), however, uncontrolled apoptosis can also cause pulmonary disease and prolong inflammation (2). The present study investigated the role of apoptosis and its relationship to necrosis during acute pulmonary inflammation caused by lipopolysaccharide (LPS).

**METHODS**. Balb/c mice were administered with LPS intratracheally and bronchoalveolar lavage (BAL) was performed. Apoptosis was determined by measuring histone-bound DNA fragments using an ELISA kit (Boehringer Mannheim), while necrosis was assessed by measuring cell lysis using lactate dehydrogenase (LDH) assay.

**RESULTS.** Treatment of mice with LPS causes a time-dependent increase in the number of inflammatory cells in the BAL fluids (Fig. 1). Control mice showed only resident alveolar macrophages with no apoptosis (Fig. 2A). In contrast, LPS-treated mice showed clear apoptosis of BAL cells (Fig. 2A). Microscopic studies confirmed the presence of apoptotic neutrophils and macrophages ingesting apoptotic bodies (result not shown). The number of apoptotic neutrophils increased concomitantly with the increase in neutrophil influx that peaked 1 day after the treatment. However, no necrosis could be detected at this early time point whereas apoptosis continued to increase and peaked at day 2. Necrosis gradually increased by day 2 and reached a maximal level by day 3 where it subsequently declined.

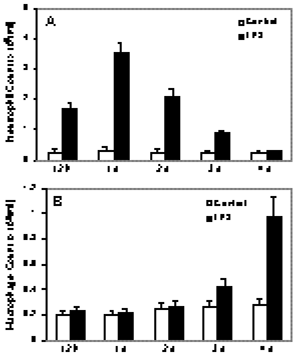

**Fig. 1.** BAL analysis of LPS-treated mice. Mice were treated with LPS (30 μg) or saline and the cell number of neutrophil (A) and macrophage (B) was determined. Data are shown as the mean ± SEM, n = 4 mice/group.

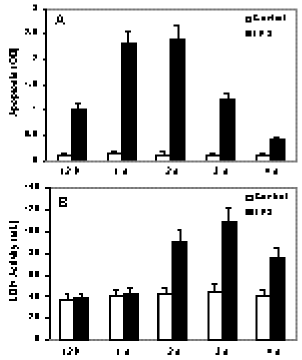

**Fig. 2.** Time-courses of apoptosis and necrosis. Mice were treated with LPS (30 μg) and BAL was analyzed at indicated times. Data are shown as the mean ± SEM, n = 4 mice/group.

**DISCUSSION**. In the present study, we demonstrated that the initial phase of lung inflammation, i.e., neutrophil influx, was not associated with necrosis but rather with apoptosis. However, necrosis developed subsequently to apoptosis, possibly due to inefficient phagocytic clearance of apoptotic cells. We conclude that the injurious effect of LPS is not a direct insult of this agent on the lung tissue but a consequence of inefficient apoptotic cell clearance that leads to the development of secondary necrosis and subsequent release of toxic contents.

**ACKNOWLEDGEMENT**. This work was supported by the National Institutes of Health Grant HL62959 (to Y.R.).

**REFERENCES.**

1. Cox, G., Crossley, J., and Xing, Z. (1995) Am. J. Respir. Cell Mol. Biol. 12, 232.
2. Hagimoto, N., Kuwano, K., Miyazaki, H., Kunitake, R., Fugita, M., Kawasaki, M., Kaneko, Y., and Hara, N. (1997) Am. J. Respir. Cell Mol. Biol. 17, 271
